# Emotion Based Attentional Priority for Storage in Visual Short-Term Memory

**DOI:** 10.1371/journal.pone.0095261

**Published:** 2014-05-01

**Authors:** Luca Simione, Lucia Calabrese, Francesco S. Marucci, Marta Olivetti Belardinelli, Antonino Raffone, Frances A. Maratos

**Affiliations:** 1 Department of Psychology, “Sapienza” University of Rome, Rome, Italy; 2 ISTC, Institute of Cognitive Sciences and Technologies, CNR, Rome, Italy; 3 ECONA, Interuniversity Center, Rome, Italy; 4 Department of Psychology, University of Derby, Derby, United Kingdom; Federal University of Rio de Janeiro, Brazil

## Abstract

A plethora of research demonstrates that the processing of emotional faces is prioritised over non-emotive stimuli when cognitive resources are limited (this is known as ‘emotional superiority’). However, there is debate as to whether competition for processing resources results in emotional superiority *per se*, or more specifically, threat superiority. Therefore, to investigate prioritisation of emotional stimuli for storage in visual short-term memory (VSTM), we devised an original VSTM report procedure using schematic (angry, happy, neutral) faces in which processing competition was manipulated. In Experiment 1, display exposure time was manipulated to create competition between stimuli. Participants (n = 20) had to recall a probed stimulus from a set size of four under *high* (150 ms array exposure duration) and *low* (400 ms array exposure duration) perceptual processing competition. For the high competition condition (i.e. 150 ms exposure), results revealed an emotional superiority effect *per se*. In Experiment 2 (n = 20), we increased competition by manipulating set size (three versus five stimuli), whilst maintaining a constrained array exposure duration of 150 ms. Here, for the five-stimulus set size (i.e. *maximal* competition) only threat superiority emerged. These findings demonstrate attentional prioritisation for storage in VSTM for emotional faces. We argue that task demands modulated the availability of processing resources and consequently the relative magnitude of the emotional/threat superiority effect, with only threatening stimuli prioritised for storage in VSTM under more demanding processing conditions. Our results are discussed in light of models and theories of visual selection, and not only combine the two strands of research (i.e. visual selection and emotion), but highlight a critical factor in the processing of emotional stimuli is availability of processing resources, which is further constrained by task demands.

## Introduction

An extensive body of literature suggests that emotional stimuli are more effective in their capture of attention than non-emotive stimuli [1]. Data from behavioural paradigms utilizing visual search, visual probe and rapid serial visual presentation (RSVP) reliably demonstrate that when there is competition for cognitive resources, emotional information, and especially that which is threatening, is typically processed more quickly and with greater accuracy than non-emotive stimuli. This is evidenced by reduced reaction times and increased accuracy of responses to such stimuli compared with non-emotive stimuli across paradigms (see [2] for a review), and suggests that emotional stimuli receive prioritised processing. This behavioural data accords well with findings from neuroimaging suggesting specific neural circuitry for the rapid and preferential processing of emotional stimuli [3], [4].

One of the most acknowledged experimental phenomena linked to competition for limited cognitive resources is the ‘attentional blink’ (AB). In a rapid serial visual presentation (RSVP) paradigm in which the AB is observed, two target stimuli are presented within a stream of distractor stimuli. If these target stimuli are presented in quick succession, e.g. 200–400 ms (or 2–4 items) apart, accurate report of the second target is impaired. This performance decrement, or AB, is thought to reflect competition between the different stimuli for attentional resources [5]. Remarkably, when the second target is motivationally relevant or an emotional stimulus, the AB is much reduced. For example, Shapiro, Caldwell & Sorensen [6] found that the AB was abolished when the second target stimulus in an RSVP stream was the participant’s own name.

More recent studies, which have utilised emotional faces (real and schematic) as target stimuli, have further demonstrated that the AB is reduced when the second target stimulus is emotional or aversive in context rather than neutral [7], [8]. This is again in line with research suggesting emotional superiority, especially for biologically prepared stimuli such as angry faces [9], [10]. In particular, Maratos, Mogg & Bradley [7], using RSVP in which the target stimuli were schematic faces depicting threatening (angry), positive or neutral facial expressions, found that performance accuracy was enhanced (i.e., the AB was reduced) on trials in which the second target was an angry face, rather than a neutral face. Such findings extend previous research by showing that angry faces reduce the AB, and that this threat-superiority competition effect is found for schematic facial expressions. However, it must be noted that whilst this effect appears to be both replicable and robust, recent AB studies have also provided evidence of a ‘happiness-superiority’ effect [11], [12] or an ‘emotion-superiority’ effect *per se* [13], [14], with some authors suggesting that *both* threatening and happy faces have a lowered threshold for identification compared with neutral faces [11], [14]. Thus whether competition results in threat superiority or emotional superiority *per se*, is still a matter of debate (as is also the idea that threatening stimuli can be processed independently of top-down attention [15]).

Competition for limited cognitive resources is also implied in visual short-term (working) memory tasks [16]. Such tasks imply both a limited processing capacity for visual stimuli and that visual short-term memory (VSTM) has a limited storage capacity. Considering this, emotional relative to neutral stimuli might further be prioritized for processing and storage in VSTM. Indeed, Jackson, Wolf, Johnston, Raymond & Linden [17] have recently demonstrated that significantly more angry face identities can be stored in VSTM than happy or neutral face identities. However, in the study reported, they presented from 1 to 4 faces of the *same* emotional category (angry, happy *or* neutral) in each trial, and used a very long memory array exposure time (i.e. 2000 ms). Such a long exposure time contrasts sharply with the short exposure time (e.g. 100–500 ms) typically used in VSTM studies [16], [18], [19]. Moreover, as Jackson et al. did not assess competition between faces of different emotional valence *within* the same trial, theoretical interpretation of findings with regard to threat and/or emotional superiority effects is limited.

It has recently been shown that spatial and temporal competition between multiple objects in VSTM and AB (RSVP) paradigms can be modelled within the *same* neural processing architecture, with involvement of the *same* processing and limited storage capacity mechanisms [20]. Consequently, analogous to AB research, it can be predicted that when stimuli have to compete for conscious awareness (i.e. limited encoding capacity) within VSTM, emotional stimuli will be prioritized. Such processing effects should be especially pronounced when competition is maximised. To expand, following Bundesen’s [16] ‘Theory of Visual Attention’ (TVA), a larger set size and reduced display exposure time lead to greater competition between visual stimuli. In TVA, this competition can be influenced by a number of factors, such as visual features (e.g., colour or shape) or category (e.g., digit or letter) of the individual stimuli. These factors change the attentional weights of the visual objects competing for limited processing capacity. Thus, it is possible to control the level of competition between simultaneously presented stimuli by modifying their presentation rate (as in AB paradigms), or their exposure time or numbers (as typical in visual search or VSTM paradigms). Therefore, when stimulus presentation time is limited and/or, more importantly, multiple objects are briefly presented, competition for the limited processing capacity of VSTM is especially high, severely limiting attentional resources available [16], [21]. This is somewhat similar to the load theory of attention and cognitive control proposed by Lavie, Hirst, De Fockert & Viding [22] (see also [23] for a review). Here it is suggested that under conditions of high perceptual load that fully engage processing capacity, there is simply no capacity for irrelevant distractor perception. Indeed, only under conditions of low perceptual load will there be resources available to process distracting (or all) stimuli. For a further discussion of visual search and attentional load see also Dosher, Han, & Lu [24].

Returning to visual attention more generally, Bundesen et al. [25] have further proposed the ‘Neural Theory of Visual Attention’ (NTVA), which assumes two stages or ‘waves’ of processing: a first wave of unselective processing, that comprises initial sensory processing, formation of perceptual units (object segmentation), and computation of attentional weights; and a second wave of selective processing, in which the attentional weights computed during the first wave are used for redistribution of cortical processing capacity across objects in the visual field. This model, whilst also similar to Lavie et al. [22], assumes a common substrate for visual competition with simultaneous [26] and sequential [27] stimulus presentation, thus linking VSTM and AB results. Interestingly, in research by Pessoa, Kastner & Ungerleider [28] a similar argument is put forward to explain the emotional superiority effect. These authors suggest that an initial volley of activation over occipitotemporal cortex takes place when both emotional and non-emotional faces are viewed, but that this is followed by a second wave of processing with signals from other brain structures (e.g. the amygdala) then converging. This second wave leads to the selection of stimuli based on their emotional valence.

To our knowledge, there have been no previous studies with manipulation of set size and/or exposure time to study competition between simultaneously presented stimuli with different emotional valences within VSTM. Rather in previous studies the emotional valence of stimuli has been kept constant within trial (e.g. all to be remembered items on a given trial have the same emotional expression, as in Jackson et al. [29]). In addition, there have been no previous studies in which schematic faces have been used as stimuli to study the effects of emotional expressions in a VSTM task. Namely, when studying the effects of emotion on VSTM, pictures of real faces (usually in change detection tasks) are employed [17], [29], [30]. Schematic faces, however, are argued to offer an unambiguous representation of the key features of emotional expressions [31]–[33], whilst controlling for potential confounds of familiarity and low-level perceptual pop-out such as conspicuous light or dark areas [28]. The latter are apparent, for example, when an individual is smiling compared with frowning. Thus, to study competition between faces of different emotional categories for storage in VSTM, and in particular to test whether threatening (compared with neutral and/or happy) faces have a preferential bias in attentional selection for storage in VSTM, we used an original VSTM target report procedure with schematic neutral and emotional (angry and happy) facial expressions as the probe and non-probe stimuli. We used the Öhman et al [33] schematic stimulus set as these stimuli have been used to good effect (i.e. reliably demonstrate emotional superiority) in previous research [7], [34]. We further displayed faces with expressions of different emotion categories within the *same* trial [cf. 17].

We hypothesised that emotional, but especially threatening faces, would have a preferential bias in attentional selection for storage in VSTM, in encoding conditions where there is much competition for limited perceptual processing resources [14], [16], [25]. Thus in Experiment 1 we manipulated the exposure duration time of the memory array, and in Experiment 2 we manipulated the number of schematic faces presented in the memory array (set size) under increased time pressure, in line with earlier (but non-emotive) studies on limited perceptual processing capacity [21] (see [16] for a review). We predicted that a report bias of emotional (threatening and happy) over neutral face stimuli would be observed in a challenging encoding condition, i.e. a short exposure time. However, we further predicted that in the most challenging condition (i.e. brief exposure with large set size) a threat, rather than emotional, report bias (i.e. threat superiority) would be observed. Under such conditions, the competition between the representations of the presented stimuli would be biased in favour of the selection of threatening faces, in line with: i) earlier AB evidence with the same stimuli [7], [34]; ii) the evolutionary survival advantage of processing this stimulus type compared with happy faces - i.e. immediate danger; and iii) the models of visual attention and cognitive load presented. To assess this, in the first experiment we compared response accuracy for a short display presentation time (high competition) with response accuracy for a long display presentation time (low competition) using a set size of four facial stimuli. In the second experiment we contrasted response accuracy for a small set size with response accuracy for a large set size while exposure time was kept fixed at the short presentation time, to maximise attentional competition in the large set size condition (i.e. high competition vs. maximal competition, respectively).

## Experiment 1

### Materials and Methods

#### Ethics statement

All individuals gave informed written consent to participate in the experiment, which adhered to the tenets of the Declaration of Helsinki and received local ethical committee approval from the Psychology Ethics Committee, “Sapienza” University of Rome.

#### Participants

Twenty participants (9 female; mean age 23.3 years, SD 1.71 years) from “Sapienza” University of Rome took part in the experiment. All participants reported no history of neurological or psychiatric disorder and had normal or corrected to normal vision. Data from one participant was excluded due to technical problems, this left a final sample of 19 participants (9 female; mean age 23.3 years, SD 1.67 years) from which data were analysed.

#### Stimuli

**Figure 1:**
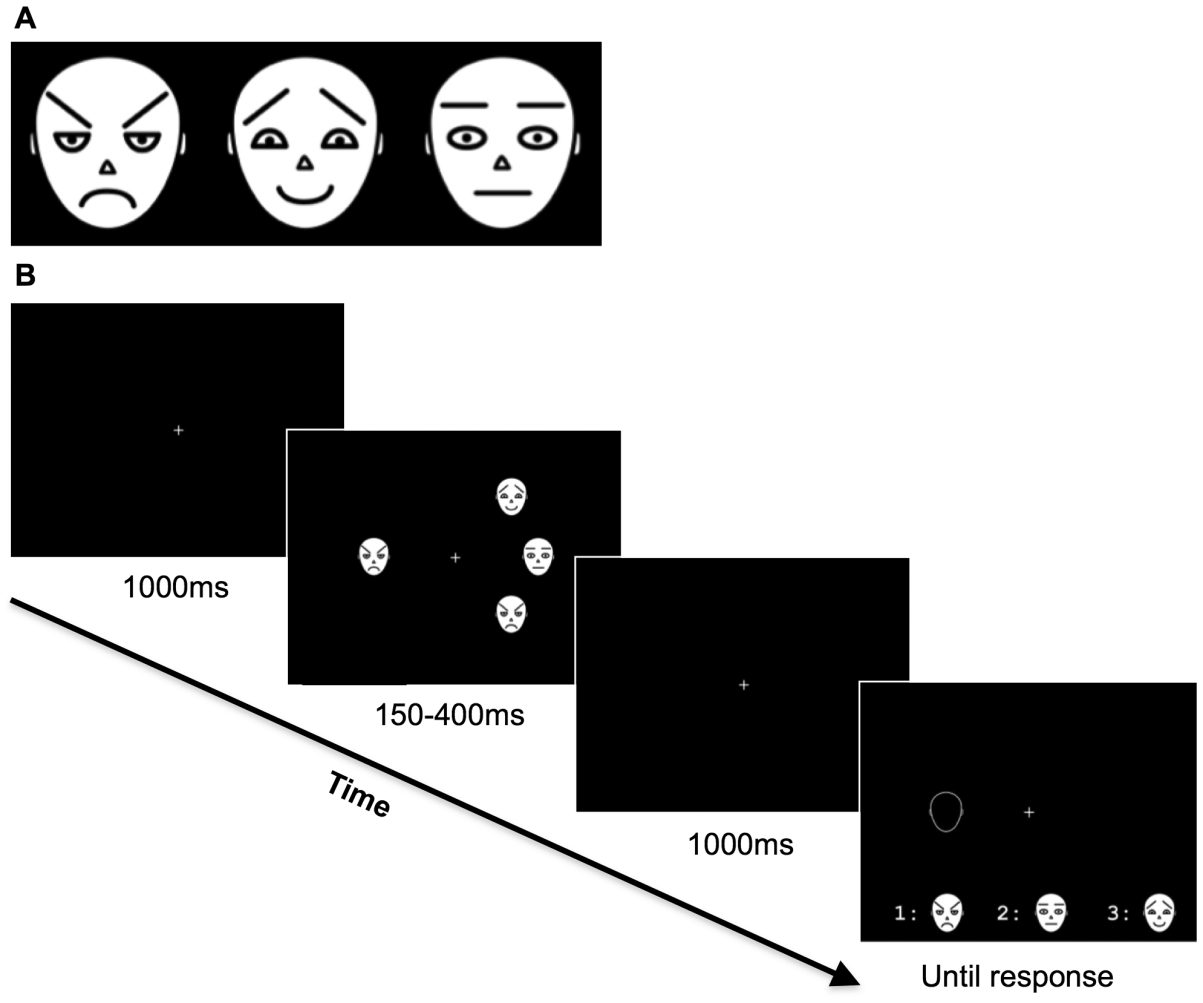
The three emotional expressions of the schematic faces used in the experiments, i.e. Angry, Happy and Neutral faces (Panel A), and an example of the sequence of events in each trial in experiment 1 (Panel B).

We used the same schematic faces as Maratos et al. [7]. These included an angry (A), a happy (H) and a neutral (N) face. Each differed with respect to three main features: eyebrow, eye and mouth shape (see Figure 1A). Adapting the VSTM task proposed by Landman, Spekreijse & Lamme [35], the stimuli were presented in one of eight possible locations around the centre of a flat screen monitor (LG 16′, 60 Hz refresh rate). Each stimulus subtended a region of approximately 2°×2.2° visual degrees, and was presented at a distance of 5°±0.5° from the centre, resulting in an average distance between any two stimuli of 3.8° visual degrees. All stimuli were white on a black background. A small fixation cross was presented on the centre of the screen throughout each trial. Stimulus presentation was controlled by E-prime software (version 1.0).

#### Procedure

In the VSTM experimental task, each trial started with the presentation of a fixation cross at the centre of the screen. Participants were instructed to fixate this cross throughout the trial. After 1000 ms the memory array was presented. This array consisted of the schematic faces, each one randomly placed in one of eight possible locations around the fixation cross. The memory array was presented for either 150 ms (‘short’ exposure time) or 400 ms (‘long’ exposure time), followed by a 1000 ms retention interval. As with most VSTM tasks, the retention interval employed was significantly longer than iconic memory duration [18]. After the retention interval, a probe (an ‘empty’ face outline) was presented at the location of one of the four presented faces. The task of the participant was to report the expression of the schematic face that had appeared at the probed location, by pressing a keyboard digit from 1 to 3 associated with the three possible facial expressions (i.e. Angry, Happy or Neutral). The probed face could be Angry, Happy or Neutral with equal probability (i.e. 1/3 of trials for each emotional expression), and the digit/face choices were presented in a row at the bottom of the screen, counterbalanced across participants (see Figure 1B). After a response was recorded, a blank screen was presented for 1000 ms, before a new trial was initiated.

Prior to the experimental session proper, participants performed 24 practice trials; four for each of the six possible combinations of Emotional Expression Probed (Angry, Happy, Neutral) and Exposure Time (150 ms, 400 ms). After this practice and following verification that the participant had understood the task, each participant completed 180 experimental trials. These consisted of 30 trials for each of the six combinations of Emotional Expression Probed and Exposure Time, divided into two blocks of 90 trials separated by a short rest interval. The trial sequence for the six different conditions was fully randomized within the two blocks, and the dependent variable was correct report of the probed stimulus (Angry, Happy, or Neutral in percentage accuracy).

The experiment was administered individually to each participant in a quiet, dark room, and the experimental session lasted circa 30 minutes.

#### Data screening and analysis

Data from trials with reaction times (RT) shorter than 200 ms or longer than 10 seconds were removed. This was less than 0.8% of the data. Post hoc comparisons were computed using t tests. Alpha levels were set at .05.

### Results

**Figure 2:**
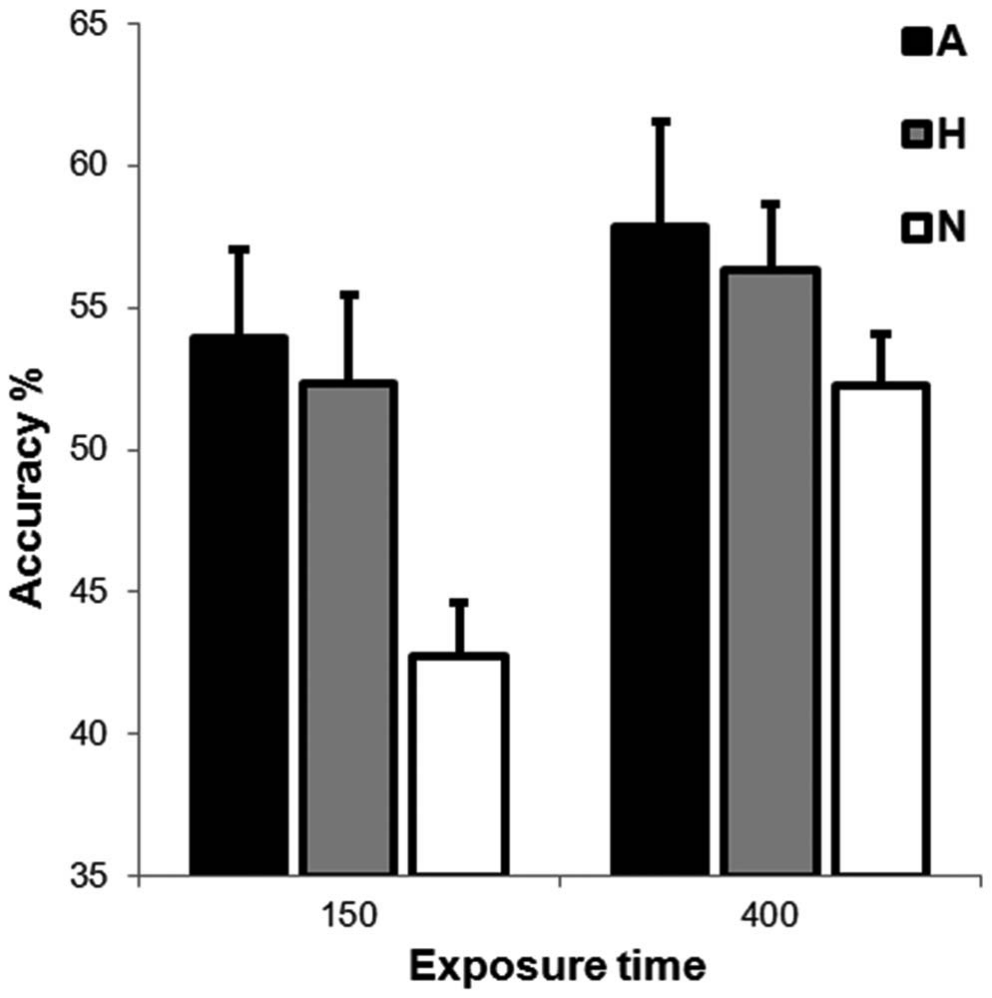
Mean percentage accuracies for the different probed faces (Angry, Happy and Neutral) and exposure times (white columns for the 150 ms exposure time, black columns for the 400 ms exposure time). Error bars represent standard error of the mean.

The mean percentage of correct report accuracy across conditions was 52.6%, with a chance level of 33.3% (i.e. participants were given three probe choices per trial, i.e. Angry, Happy, or Neutral). Figure 2 shows the mean percentage of correct report accuracy as a function of Emotional Expression and Exposure Time. A 2×3 repeated measures ANOVA of percentage report accuracy with Exposure Time (150 ms, 400 ms) and Emotional Expression (Angry, Happy, Neutral) as within-subjects factors revealed both main effects of Emotional Expression, *F* (1,18) = 11.24, *MSE* = 0.015, *p*<.01, and Exposure Time, *F* (2,36) = 5.07, *MSE* = 0.009, *p*<.05, but no interaction, *F* (2, 36) = 0.96, *MSE* = 0.01, *p* = .39.

For the main effect of Exposure Time it was evident that accuracy was greater for the 400 ms exposure time (55.5%) as compared to the 150 ms exposure time (49.7%), *p*<.01. For the main effect of emotional expression, accuracy did not differ between the angry (55.9%) and happy faces (54.3%), *p* = .60, but accuracy for the neutral faces (47.5%) was significantly lower than accuracy for the angry faces, *p*<.01, and the happy faces, *p*<.05.

To directly test our *a priori* hypotheses, we further conducted planned comparisons for the emotional versus neutral conditions at both the 400 ms and the 150 ms exposure times. The contrasts revealed significant differences only for the short exposure time (150 ms). Here, correct report accuracy for neutral faces (42.7%) was significantly lower than that for either angry (53.9%), *p*<.01, or happy faces (52.3%), *p*<.05. Of importance, accuracy for angry faces did not differ from that of happy at 150 ms, *p* = .70.

### Discussion

Based on previous research demonstrating that: i) the AB effect is reduced when the second target stimulus is emotive or aversive in content [7], [8], [11], [14], [36]; ii) there is overlap of selective processing mechanisms in AB and VSTM tasks [20]; and iii) the processing capacity of visual information is limited [16], [22], [24], we predicted an emotional superiority effect for the storage of schematic face stimuli in VSTM under the constrained presentation time condition. That is, we predicted higher report accuracy for both angry (i.e. threatening) and happy faces versus neutral faces in the VSTM task, especially at the short exposure time.

Consistent with this, we found: i) an overall selection and encoding superiority effect for the emotional compared with neutral faces; and ii) greater storage of the threatening and happy, compared with neutral, schematic faces at the short exposure time. Thus our findings are compatible with literature suggesting that emotional stimuli capture and hold attention in a manner unlike that of non-emotive stimuli. Indeed it is has been argued that both threatening and happy faces have a lowered perceptual threshold for identification than neutral faces (as evidenced by recent AB emotion superiority findings [11], [14]), because both types of expression play a crucial role in interpersonal communication [37].

To expand, when increasing task demands by decreasing exposure time from 400 to 150 ms, our planned comparisons revealed that both the angry and happy faces were prioritised for storage. This was evidenced by their increased report accuracy in comparison to the neutral faces. Whilst the effects of threat superiority are well documented, ‘happiness-superiority’ effects are less well documented, but have been suggested to reflect the ease at which such stimuli are perceived (i.e. perpetual saliency [11], [14]) as well as the idea that happy faces broaden attention. That is, Srivastava & Srinivasan [12] (see also [38]) suggest that happy faces require fewer processing resources than negative stimuli (such as sad faces). Thus in the present experiment both angry and happy faces ‘survived’ the relative competition effects. This said, de Jong et al. [14] further argue that when angry and happy face stimuli compete for limited cognitive resources (such as within a limited time period with high task demands), processing priority is assigned to the threatening face only. This is consistent with the idea that threat-related biases only emerge when competition is maximal (see also [39]).

Given an estimated VSTM capacity of about four objects [16], [18], [20], [25], [40], it can further be argued that it was not the limited storage capacity of VSTM that prevented storage of the probed faces. Rather, our evidence suggests that a perceptual processing limitation combined with a short display exposure prevented encoding and storage of the probed faces in VSTM [16]; indeed, even with the longer exposure duration of 400 ms performance was poor; i.e. we found an average percentage accuracy of around 55%. However, to increase competition further, in Experiment 2 we manipulated the set size of the memory array, while keeping display array time constant (but at the constrained presentation time of 150 ms). Here, and in line with both theoretical models and previous literature pertaining to the threat superiority effect, we predicted a preferential processing and attentional selection bias for the angry faces only.

## Experiment 2

### Materials and Methods

#### Ethics statement

All individuals gave informed written consent to participate in the experiment, which adhered to the tenets of the Declaration of Helsinki and received local ethical committee approval from the Psychology Ethics Committee, “Sapienza” University of Rome.

#### Participants

Twenty participants (mean age = 26.7; SD = 5.1; 9 females) from “Sapienza” University of Rome took part in the experiment. As in Experiment 1, all participants reported no history of neurological or psychiatric disorder, and had normal or corrected to normal vision.

#### Stimuli

The stimuli were the same as used in experiment 1.

#### Procedure

The procedure was the same as described in experiment 1, with the exception that set size was manipulated rather than array display time. That is, the array consisted of either three or five schematic faces, each one randomly placed in one of eight possible locations around the fixation cross for a fixed duration of 150 ms. The probed face could be Angry, Happy or Neutral with equal probability (i.e. 1/3 of trials for each emotional expression) while the emotional expression of the other faces was fully randomised. As in experiment 1, the digit/face choices were presented in a row at the bottom of the screen counterbalanced across participants, and participants performed 24 practice trials: four for each of the six possible combinations of Emotional Expression Probed (Angry, Happy, Neutral) and Stimulus Set Size (three stimuli, five stimuli). After this practice and following verification that the participant had understood the task, each participant completed the 180 experimental trials. These consisted of 30 trials for each of the six conditions, divided into two blocks of 90 trials separated by a short rest interval. The trial sequence for the six different conditions was fully randomized within the two blocks.

#### Data screening and analysis

As for the first experiment, data from trials with reaction times (RT) shorter than 200 ms or longer than 10 seconds were removed. This was less than 0.7% of the data. Post hoc comparisons were computed using t tests. Alpha levels were set at .05.

### Results

**Figure 3:**
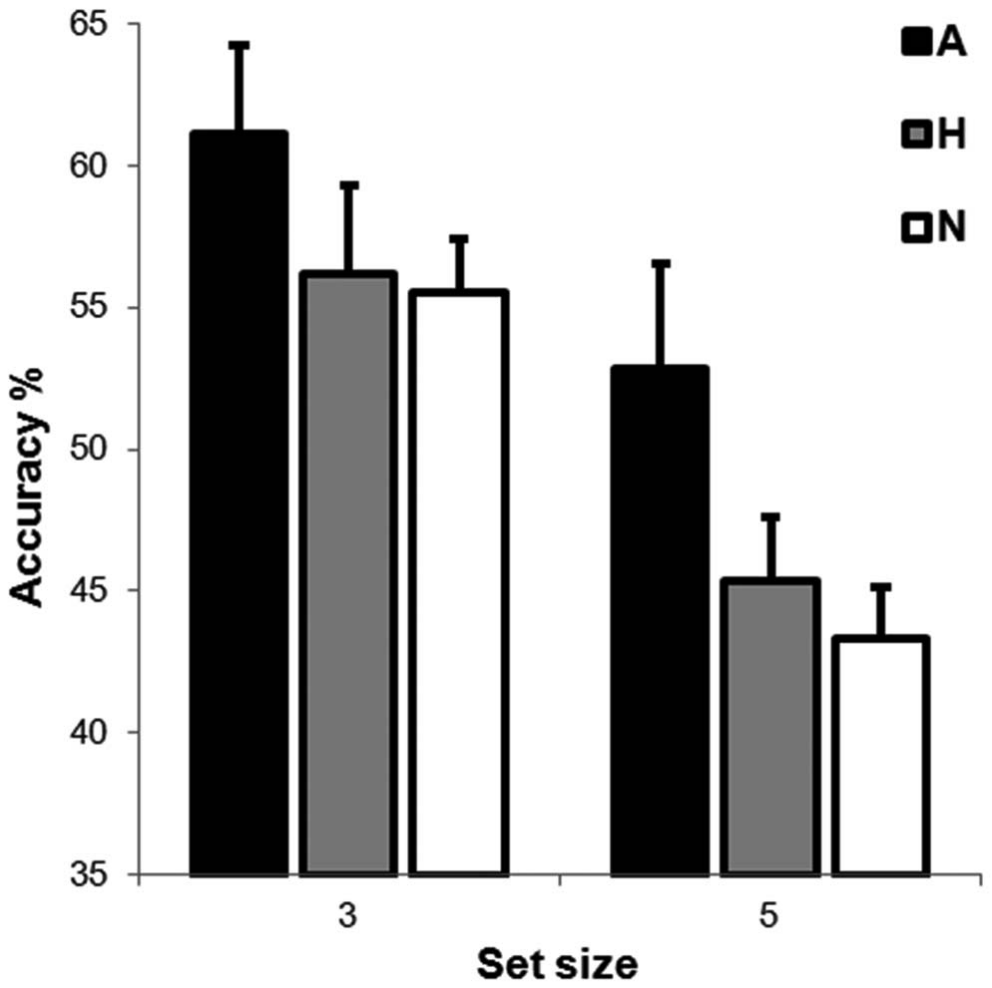
Mean percentage accuracies for the different probed faces (Angry, Happy and Neutral) and set size (white columns for set size 3, black columns for set size 5). Error bars represent standard error of the mean.

The mean percentage of correct report accuracy across conditions was 52.4% (chance level = 33.3%). Figure 3 shows the mean percentage of correct report accuracy as a function of Emotional Expression and Set Size. A 2×3 repeated measures ANOVA of percentage accuracy with Set Size (three stimuli, five stimuli) and Emotional Expression (Angry, Happy, Neutral) as within-subjects factors revealed a main effect of Set Size, *F* (1, 19) = 28.7, *MSE* = 0.01, *p*<.001, and a main effect of Emotional Expression, *F* (2, 38) = 3.82, *MSE* = 0.017, *p*<.05. The analysis revealed no interaction effect between the two variables, *F* (2, 38) = 0.48, *MSE* = 0.008, *p* = .62.

For the main effect of Set Size it was evident that accuracy was greater when three faces were presented (57.6%) compared with when five faces were presented (47.2%), *p*<.001. For the main effect of Emotional Expression, accuracy for the angry faces was significantly higher than accuracy for the happy faces, *p*<0.05 (56.9% vs. 50.8% respectively), and for the neutral faces, *p*<.05 (56.9% vs. 49.4% respectively), but accuracy did not differ between the happy and neutral faces, *p* = .67 (50.8% vs. 49.4% respectively).

To directly test our *a priori* hypotheses, we further conducted planned comparisons between the report accuracies for the three emotional expressions, within each set size. The contrasts revealed significant differences only for set size five. Of importance, here, performance accuracy on both happy (45.4%) and neutral (43.3%) probe trials was significantly worse than performance accuracy on angry probe trials (52.8%), *p*<.05 in both cases. All other contrasts were not significant.

### Discussion

In this second experiment, we maximised stimulus competition by manipulating set size whilst keeping presentation time fixed at the shorter (constrained) duration. Given the greater competition between face representations in the visual system with a larger set size combined with the additional time exposure constraint, in this experiment we hypothesised attentional prioritisation of threatening faces over *both* neutral and happy faces for a set size of five stimuli. In accordance with this hypothesis, greater accuracy was observed for the angry probed faces compared with both the neutral and happy probed faces. This was plausibly due to the limited storage capacity of VSTM combined with the limited perceptual processing capacity. This said, it should be noted that even with a small set size in this experiment, the average percentage accuracy for this small set size was around 57%, thus reflecting a remarkable influence of perceptual processing limitations. This finding, as well as the threat compared with emotional superiority effect found here, is discussed in greater detail below.

## General Discussion

To investigate whether emotional and in particular threatening faces have priority for storage in visual short-term memory (VSTM) with enhanced competition between object (face) representations, we performed two experiments with the presentation of angry, happy and neutral schematic faces to be stored in VSTM. In the first experiment we manipulated display exposure time (whilst set size was kept constant), and in the second experiment we manipulated set size (while exposure time was kept constant at the shorter duration). We predicted that competition between face representations would occur when a short (constrained) exposure time was utilised, but that competition would be maximal when a short exposure time combined with a large set size was utilised. Thus related to the different levels of competition, we hypothesised emotional superiority and threat superiority, respectively, due to the combination of different attentional weightings as a consequence of task demands. Our findings were consistent with hypotheses. That is, we found an emotional superiority effect in VSTM storage when a brief exposure duration was utilised, but only a threat superiority effect in VSTM storage when a brief exposure time combined with a large set size was utilised.

In explaining this result, we argue that in a VSTM task with a shorter exposure duration combined with a larger set size, the increasingly brief sensory input *together* with competition among multiple object representations necessarily biases attention in perceptual processing and/or prevents consolidation for storage in VSTM [16], [22], [25], [41]. Here therefore, only stimuli of the greatest survival significance (i.e. that which is biologically prepared to initiate a fight or flight response, see [9] [10]) would likely produce activation of sufficient strength and duration required for consolidation and storage in VSTM, thus explaining differences in attentional prioritisation of VSTM across our two experiments. That is, in experiment 1, we found a VSTM storage bias for both angry and happy faces over their neutral counterpart (consistent with AB research by Miyazawa & Iwasaki [11] and de Jong et al. [14]). However, under situations of maximal competition, the combined pressures of limited display duration and large set-size ensured competition between our emotive stimuli and, as such, the emergence of threat-superiority (consistent with AB research by Maratos et al. [7]). To expand, the apparent disappearance of the happy face effect in Experiment 2 under maximal task pressure reveals that the increased level of competition allowed only the ‘survival’ of the strongest stimuli, i.e. the threatening faces; even if under more relaxed task constraints (as in experiment 1) emotional superiority (and the happy face effect) emerged. Put another way, and considering load theory [22], the high perceptual load in our final condition rendered all but the most essential stimuli (i.e. angry faces) irrelevant.

In addition, our present findings are generally consistent with previous VSTM experiments utilising change detection paradigms [17], [29], [30]. Whilst in these studies real (as opposed to schematic) faces were used, all reported an advantage for angry or threatening faces over neutral faces. Interestingly, in these previous experiments the threat superiority effect emerged even with a 2000 ms exposure time [17], or small set size of one or two faces [42]. These discrepant results could be explained by the different stimuli used in the experiments: real faces are far more complex stimuli than schematic faces, thus leading to greater competition between object representations even with limited stimuli or a very long display exposure time. Moreover, with real faces, the estimated number of faces stored in VSTM is often less than one, even when two or more faces are presented [30], [42]. In other words, no more than one ‘real’ face can be stored in VSTM at one time, even if the VSTM capacity for simple visual stimuli has been estimated at about four objects [18]. This again demonstrates the importance of competition (or load) when considering attentional and memory constraints for stimulus, and especially emotional face, processing.

Of importance, our findings can also be interpreted in light of Bundesen et al.’s [25] NTVA two stages or ‘waves’ of processing model. This model assumes a first wave/stage of unselective processing and a second stage/wave of selective processing. It also accords well with research by Pessoa, Kastner & Ungerleider [28] who suggest that initially emotional and neutral faces are undifferentiated (i.e. they are processed as equivalents), and that it is only after a second wave of processing, involving structures such as the amygdala, that the selection of stimuli based on their valence emerges. This hypothesis is consistent with results from event-related potential studies [43], [44], revealing a late effect of emotional valence on face processing over the occipital cortex at around 250–300 ms; i.e. after initial face recognition (circa 170 ms) as indicated by the N170 [45]. As an alternative, however, given that more recent evidence demonstrates very early activation of the amygdala in response to emotional (and especially threatening) stimuli [4], [46], [47], it could be argued that in certain situations the first wave takes place extremely rapidly. That is, in cases of imminent threat the amygdala (and/or related structures) *rapidly* signals to occipital and occipitotemporal cortices allowing for the early redistribution of attentional weights to objects in the visual array [47], [48].

Combining NTVA with the above views to account for our experimental evidence, it can be argued that the presentation of a visual array comprising schematic faces with emotional expressions would elicit a wave of processing in which the face features are processed in occipitotemporal cortex circa 50–200 ms from stimulus presentation. Then, the attentional weights are computed for the second processing wave, in which processing is biased in favour of emotional faces, and in particular those which are threatening. Our experimental evidence suggests that exposure time and set size modulate the competition between representations for the available processing resources. Under moderate competition conditions, as in the brief exposure time condition of experiment 1, both the threatening and positive stimuli are likely to be stored in VSTM. Under maximal competition (i.e. a large set size and brief exposure time, as in experiment 2), however, only the most ecologically salient stimuli maintain sufficient attentional weight to ensure storage in VSTM. This relates to the disappearance of the happy face effect in this more demanding processing condition. Of importance this is not automatic but dependent upon attentional/task constraints, as evidence by the fact that in neither of our experiments performance was at ceiling (i.e. 53% and 52%, in experiment 1 and 2 respectively). Certainly, if the threat stimuli were processed automatically (i.e. without requiring attention), we would have found performance for angry faces near ceiling. Instead, our data suggest that angry faces also needed to compete for processing resources, but that they received a strong attentional weight linked to their survival importance and hence biased competition in their favour.

This finding is consistent with the work of Pessoa, McKenna, Gutierrez & Ungerleider [49], who demonstrated that emotional superiority (as well as the threat effect) depends upon sufficient attentional resources being available for the processing of emotional facial expressions; that is, such stimuli are prioritised but *not* processed automatically (see also [9]). Consistent with this, in a flanker experiment utilising schematic faces with different emotional expressions as the flankers, Barratt & Bundesen [50] also found that attentional capture by angry faces was not automatic, but depended on attentional settings. Taken together, this experimental evidence and theory combined, converge on the view that a critical factor in the processing of emotional stimuli is the amount of available processing resources [cf. 15].

Our present findings can also be accounted for by the recent Visual Selection and Awareness (ViSA) model [20], which already offers plausible explanations for a large number of attentional blink and VSTM findings. Indeed, in ViSA, the consolidation for storage in VSTM depends on the availability and strength of the perceptual representations of target objects. According to ViSA, to be consolidated and encoded in VSTM, the (neural) representations of visual objects (including schematic faces) need to be activated with sufficient strength and duration, with a longer consolidation time required for visual objects with shorter or weaker sensory inputs, such as with visual masking. This implies that if sensory input is brief or masked, targets may not be encoded, or may be encoded with a slower consolidation in VSTM, due to weaker perceptual representations supporting consolidation. Thus, according to ViSA, the (neural) representations of schematic faces would be less activated with a shorter exposure time (as in Experiment 1), or with enhanced competition (mutual inhibition) due to a brief exposure time combined with a larger set size (as in Experiment 2). This dynamics in perceptual processing would influence higher-level processing for consolidation of target-related information, by slowing down or preventing storage in VSTM. The activation of threatening face representations would however be enhanced by an attentional weighting mechanism (described above), thus counteracting shorter sensory input availability or enhanced inhibition linked to the presentation of other faces.

This said, a weakness of our present experiments was the lack of control of the non-probed stimuli, as we manipulated in our experiments only the emotional expression of the probed stimulus. By controlling the number and the type of all stimuli in the memory array, we would be able to study with greater accuracy how emotional expressions compete with one another to access the ‘limited visual processing capacity’ for storage in VSTM. With such a procedure, it would be possible to compare conditions in which all the stimuli receive the same attentional weights (congruent condition), with conditions in which stimuli receive different attentional weightings (e.g. incongruent and/or mixed conditions). This would allow for a more systematic investigation of attentional prioritisation for storage in VSTM, as well as comparison to related attentional paradigms traditionally used to investigate emotion superiority such as visual search. In the latter, participants must fixate a central fixation and stimuli can appear at concentric locations, as in the present VSTM paradigm (see for example [33]).

In addition, for a further investigation of the time course of attentional bias for threatening faces in a VSTM task, a masking procedure could be used. In particular, with the experimental settings used in this paper, the presentation of a visual mask after a variable lag from memory array offset could be used, as in Vogel, Woodman & Luck [51]. This procedure would prevent any iconic memory contribution to visual processing after memory array offset, and allow a more refined investigation of the time-course of emotion-related attentional weighting in VSTM. It can further be hypothesised that a VSTM threat superiority effect would be even more marked in anxious participants, plausibly due to greater feedforward and/or feedback amplification via the amygdala [52], [53]. Such a hypothesis can be straightforwardly tested in a behavioural investigation with our present experimental paradigm. Moreover, biologically-plausible computational modelling of storage in VSTM can be incorporated to account for the present results. For example, the ViSA model [20] could be further developed with an amygdala module providing input to visual cortex (both feedforward and feedback), which allows for attentional weighting and/or a saliency map to bias competition between object (face) representations in perceptual processing for storage in VSTM.

To sum, we have demonstrated that emotional prioritisation in VSTM depends upon the competition prevalent between items (over both space and time) and hence task demands. We argue that emotional face processing is not automatic but depends upon the specific competition pressures. Under moderate competition emotional superiority is observed whereas under maximal competition threat superiority is observed. This finding accords well with previous emotional research (both behavioural and neuroimaging) and theory, and can be explained by models of visual selection; thus combining the two strands of research.
